# Interactions Between Opioids and Dextroamphetamine on Locomotor Activity: Influence of an Opioid's Relative Efficacy at the Mu Receptor

**DOI:** 10.3389/fpsyt.2021.790471

**Published:** 2022-01-05

**Authors:** Mark A. Smith, Shannon L. Ballard, Clarise F. Ballesteros, Samantha A. Bonge, Alexander T. Casimir, Lauren M. Childs, Max A. Feinstein, Annie K. Griffith, Alexandra N. Johansen, Daegeon Lee, A. Caroline Mauser, Cassidy M. Moses, Ian J. Robertson, Javier U. Robles, Justin C. Strickland, Mary E. Walters, Seeley J. Yoo

**Affiliations:** Program in Neuroscience, Department of Psychology, Davidson College, Davidson, NC, United States

**Keywords:** addiction, drug interaction, drug combination, pharmacotherapy, polydrug abuse

## Abstract

Opioids and stimulants are often used in combination for both recreational and non-recreational purposes. High-efficacy mu opioid agonists generally increase the behavioral effects of stimulants, whereas opioid receptor antagonists generally attenuate the behavioral effects of stimulants; however, less is known regarding the interactions between stimulants and opioids possessing low to intermediate efficacy at the mu receptor. The purpose of this study was to examine the role of an opioid's relative efficacy at the mu receptor in altering the behavioral effects of dextro(*d*-)amphetamine. To this end, opioids possessing a range of relative efficacy at the mu receptor were examined alone and in combination with cumulative doses of *d*-amphetamine on a test of open-field, locomotor activity in male rats. Levorphanol, buprenorphine, butorphanol, nalbuphine, (-)-pentazocine, (-)-metazocine, (-)-cyclazocine, (-)-NANM, and nalorphine increased the locomotor effects of *d*-amphetamine in either an additive or greater-than-additive manner according to an effect-additive model. Only the selective, high-efficacy kappa agonist, spiradoline, and the non-selective opioid receptor antagonist, naloxone, failed to increase the effects of *d*-amphetamine under the conditions examined. These data indicate that opioids possessing a large range of relative efficacy at the mu receptor, including those possessing very low relative efficacy, significantly increase the locomotor effects of *d*-amphetamine.

## Introduction

Opioids and stimulants are often used in conjunction for both recreational and medicinal purposes. For instance, prescription and non-prescription stimulants are sometimes used in combination with licit and illicit opioids under recreational conditions to increase the euphorigenic effects and decrease the aversive effects of the other compound (1, 2). Human laboratory studies report that stimulant-opioid combinations produce subjective effects of greater intensity than either drug alone [(3–7)], and preclinical animal studies report that stimulant-opioid combinations are selected more often than either drug alone in concurrent choice procedures (8, 9). Opioids are used extensively for both acute and chronic pain conditions, whereas amphetamines are widely used in the clinical management of medical disorders such as obesity and attention-deficit hyperactivity disorder. Importantly, these types of conditions often co-occur with one another, and it is not uncommon for an individual to use prescription opioids and amphetamines simultaneously (10–12). Given the frequency with which these drugs are co-administered in both recreational and clinical settings, it is important to understand the pharmacological mechanisms determining their interactions.

One factor determining the interactions between opioids and stimulants is an opioid's relative efficacy at mu receptors. Opioids vary in their selectivity for and efficacy at the three primary opioid receptors (mu, kappa, delta), and these pharmacological properties determine their qualitative and quantitative effects when combined with stimulants. For instance, opioids with high efficacy at the mu receptor (i.e., full mu agonists) typically increase the effects of cocaine, dextroamphetamine (*d-*amphetamine), and other stimulants (13, 14), whereas opioids with high efficacy at the kappa receptor (full kappa agonists) and opioids with null efficacy at the mu receptor (i.e., mu opioid antagonists) typically decrease or block the effects of stimulants (15–17). Opioids with low to intermediate relative efficacy at the mu receptor (i.e., partial mu agonists) may increase or decrease the effects of stimulants, depending on the assay, dependent measure, and experimental parameters [c.f. (18–27)]. For instance, we previously reported that intermediate-efficacy opioids with a large range of relative efficacy at the mu receptor (e.g., buprenorphine, butorphanol, nalbuphine) increase the effects of cocaine on locomotor activity, and only opioids with very low relative efficacy at the mu receptor (e.g., nalorphine) fail to increase cocaine's locomotor effects (28). Cocaine is a dopamine reuptake inhibitor, and it is not known whether intermediate-efficacy opioids produce similar effects when combined with stimulants possessing other mechanisms of action (e.g., promoting dopamine release).

The purpose of this study was to examine the effects of opioids possessing a range of relative efficacy at the mu receptor on locomotor activity induced by *d-*amphetamine, a monoamine releaser with a high affinity for the dopamine transporter. To this end, various doses of opioids were examined alone and in combination with cumulative doses of *d-*amphetamine in a test of open-field, locomotor activity. The opioids tested varied in their relative efficacy at the mu receptor, with an estimated rank order of levorphanol > buprenorphine > butorphanol ≥ nalbuphine > (-)-metazocine ≥ (-)-pentazocine ≥ (-)-cyclazocine (29–31). The selective high-efficacy kappa agonist, spiradoline, and the non-selective opioid receptor antagonist, naloxone, served as negative controls. We tested the hypothesis that an opioid's ability to enhance the effects of *d-*amphetamine would vary directly with its relative efficacy at the mu receptor.

## Materials and Methods

### Subjects

Male, Long-Evans rats were obtained from Charles River Laboratories (Raleigh, NC, USA) and weighed ~280 g upon arrival. Subjects were housed individually in transparent cages in a colony room maintained on a 12-h light/dark cycle (lights on 0500). Subjects were maintained at 300–350 g during behavioral testing *via* light food restriction. Drinking water was available *ad libitum* in the home cage, and environmental enrichment (e.g., bedding, gnaw sticks, plastic tubes) was provided throughout the study. All rats were tested and maintained in accordance with the guidelines of the Institutional Animal Care and Use Committee of Davidson College and the *Guide for the Care and Use of Laboratory Animals* (32). A total of 119 rats were divided between 12 groups: time-course (*n* = 21; *n* = 5–6/dose), levorphanol (*n* = 10), buprenorphine (*n* = 9), butorphanol (*n* = 10), nalbuphine (*n* = 9), (-)-pentazocine (*n* = 10), (-)-metazocine (*n* = 10), (-)-cyclazocine (*n* = 10), (-)-NANM, nalorphine (*n* = 10), spiradoline (*n* = 10), and naloxone (*n* = 10).

### Materials

All behavioral tests were conducted in an open-field, locomotor activity chamber. The interior of the chamber was made of plywood, measured 50 x 50 x 40 cm, and painted white with high-gloss paint. The lid of the chamber was made of transparent Plexiglas, which allowed all activity to be monitored by a video camera suspended 1.5 m above the apparatus. Heavy black lines were drawn on the lower surface of the apparatus with indelible ink that could easily be observed from the camera mounted above. These lines divided the floor into a grid of 25 squares, each measuring 10 x 10 cm. A wire-mesh screen was permanently suspended 2 cm above the bottom of the apparatus and served as the floor of the apparatus during behavioral testing.

### Behavioral Procedure

Prior to behavioral testing, rats in each group were habituated to the testing environment by being placed into the activity chamber for 300 s a day for five consecutive days. After these initial habituation sessions, non-injection control tests were conducted in which locomotor activity was measured across multiple observation periods. During these control tests, each rat was removed from its home cage and placed into the activity chamber for 130 s and the number of locomotor activity counts was recorded (see section Data Analysis). The first 10 s of this interval served as an acclimation period, and thus only data obtained during the final 120 s of the interval were used for statistical analysis. Immediately after the observation period, the rat was removed from the chamber and returned to its home cage. Fifteen minutes later, the rat was again placed into the chamber and locomotor activity was again measured. All control sessions continued for two additional intervals (i.e., a total of four intervals), with 15-min intervals separating each interval. Each rat received only one non-injection control session.

### Drug Administration and Locomotor Activity Testing

The effects of *d-*amphetamine were examined under a cumulative dosing procedure. In this procedure, each rat was initially injected with saline and returned to its home cage. After a 15-min pretreatment interval, the rat was placed into the activity chamber for 130 s and the number of locomotor activity counts was recorded. Again, the first 10 s of the interval served as an acclimation period and only data from the final 120 s were used for statistical analysis. After the observation period had elapsed, the rat was removed from the chamber, administered the lowest dose of *d-*amphetamine, and returned to its home cage. Fifteen minutes later the rat was again placed into the chamber and locomotor activity was again measured. Each test session continued for two additional intervals, with increasing doses of dextroamphetamine administered at the beginning of each subsequent interval. Cumulative doses of 0.18, 0.56, and 1.8 mg/kg dextroamphetamine were tested in all sessions.

### Drug Combination Testing

In separate groups of rats, drug combination tests were conducted in which various opioids were administered in combination with *d-*amphetamine. Testing procedures were identical to those described above, with the exception that a selected dose of an opioid was administered during the first interval of the session in lieu of saline. Two doses of each opioid were examined in a randomized order, with a minimum of 5–7 days separating each session. In subjects tested with levorphanol, spiradoline, and naloxone, cumulative doses of *d-*amphetamine were tested alone, both before and after drug combination tests, to determine the stability of the dose-effect curve with repeated testing. Doses of test drugs were selected on the basis of a previous study in which these opioids were combined with cocaine in tests of locomotor activity [(28); levorphanol: 0.3, 3.0 mg/kg; spiradoline: 1.0, 10 mg/kg; naloxone: 0.1, 10 mg/kg; buprenorphine: 0.03, 0.1 mg/kg; butorphanol: 0.1, 0.3 mg/kg; nalbuphine: 0.3, 1.0 mg/kg; (-)-pentazocine: 1.0, 3.0 mg/kg; (-)-metazocine: 1.0, 3.0 mg/kg; (-)-cyclazocine: 1.0, 3.0 mg/kg; (-)-NANM: 3.0, 10 mg/kg; nalorphine: 1.0, 3.0 mg/kg].

### Time Course Testing

A series of time-course tests was conducted to measure the time to peak effect and duration of action of *d*-amphetamine. In these tests, different doses of *d*-amphetamine (0.18, 0.56, 1.8 mg/kg) or saline (1 ml/kg) were administered at the beginning of the session, and locomotor activity was measured 5, 15, 30, 60, and 120 min later. Non-injection control sessions were not conducted for time-course testing.

### Drugs

Dextroamphetamine hemisulfate salt, levorphanol tartrate, buprenorphine hydrocholoride, butorphanol tartrate, nalbuphine hydrochloride, nalorphine hydrochloride, naloxone hydrochloride, and spiradoline mesylate were obtained from Sigma Chemical Co. (St. Louis, MO, USA). (-)-Pentazocine, (-)-metazocine, and (-)-n-allylnormetazocine were a gift from Dr. Mitchell Picker. All compounds were dissolved in saline and administered *via* intraperitoneal injection in a volume of 1.0 ml/kg of body weight.

### Data Analysis

Locomotor activity was scored by observers who were blind to the study's hypotheses. Activity counts were measured by counting the number of instances in which a rat entered a new 10 cm x 10 cm square during the 120-s observation period. Entrances were counted only if the rat crossed the grid line marking the perimeter of the square with both forepaws. Only horizontal line crossings were measured; stereotypies and pattern of movement were not recorded. Except for the time-course tests, locomotor activity was expressed as % non-injection control, with each rat serving as its own control. These non-injection control values were calculated individually for each rat by dividing the number of activity counts observed during an interval of a test session by that obtained in the corresponding interval of the non-injection control session, and then multiplying by 100. Drug interaction data were analyzed *via* two-way, repeated-measures ANOVA, with dose of *d*-amphetamine and opioid pretreatment serving as repeated measures. Time-course data were also analyzed via repeated-measures ANOVA, with time serving as a within-subjects factor and dose of *d-*amphetamine serving as a between-subjects factor. Locomotor activity counts during non-injection control tests were analyzed across intervals via one-way, repeated-measures ANOVA. Similarly, the effects of each opioid administered alone (as determined during the first interval of drug combination tests) were examined via one-way, repeated-measures ANOVA. *d*-Amphetamine was tested alone on two occasions in groups tested with levorphanol, spiradoline, and naloxone. These tests of *d*-amphetamine alone were conducted before (Day 1) and after (Day 21) drug combination tests to determine whether repeated testing altered the locomotor effects of *d*-amphetamine. These data were analyzed via two-way, repeated-measures ANOVA using dose and day as factors.

To characterize the effects of each dose of opioid in combination with *d*-amphetamine, an effect-addictive model was used. Tests of additivity were conducted using a two-way, repeated-measures ANOVA comparing the observed effects of the combination to that that predicted by an effect-additive model using dose of *d*-amphetamine and model (observed vs. predicted) as within-subject factors. The predicted effects were calculated for each rat and each dose of *d*-amphetamine by the following formula (all values depicted as % non-injection control):


$$\begin{array}{l} \text{predicted effect = }(\text{observed effect of opioid alone }\\ \text{ − observed effect of vehicle})\;\\ \text{ + observed effect of }d\text{-amphetamine alone} \end{array}$$

The null hypothesis (i.e., the interaction conformed to an effect-additive model) was rejected if a significant main effect was obtained for the model factor.

## Results

### Time Course

**Figure 1:**
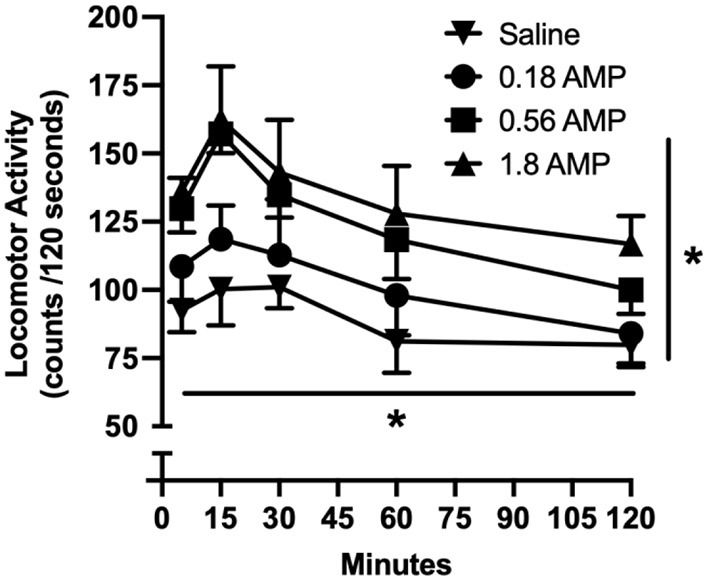
Time-course effects of acute doses of *d*-amphetamine on locomotor activity. Vertical axis reflects locomotor activity expressed as raw activity counts over 120-s observation period. Horizontal axis reflects time after administration in minutes. All data points reflect the mean of 5–6 rats. Vertical lines represent the SEM; where not indicated, the SEM fell within the data point. Asterisk with horizontal line indicates significant effect of time. Asterisk with vertical line indicates significant effect of amphetamine dose.

Locomotor activity as measured by raw activity counts increased as function of *d*-amphetamine dose and varied as function of time (Figure 1). Acute doses of *d*-amphetamine (0.18, 0.56, and 1.8 mg/kg) dose-dependently increased locomotor activity [main effect of dose: *F*_3, 7_ = 5.162, *p* = 0.010], with 0.56 and 1.8 mg/kg significantly increasing locomotor activity relative to saline (*p* = 0.030 and *p* = 0.007, respectively). Locomotor activity peaked 15 min after administration [main effect of time: *F*_1, 17_ = 45.943, *p* < 0.001]; locomotor activity counts were significantly greater at this time point than the 60- and 120-min time points (*p* < 0.001 for both time points).

### Non-injection Control

Raw locomotor activity counts obtained during the non-injection control sessions varied across groups (Table 1). This was expected given that each group of rats was obtained from separate cohorts over an 8-year period, and some genetic drift in the stock population may have occurred (e.g., baseline locomotor activity generally increased over the 8-year period). There was some variability across intervals, but this was not significant in 8 of the 10 groups tested. In groups tested with butorphanol and nalbuphine, locomotor activity counts significantly decreased across intervals of the session [main effect of interval: *F*_3, 27_ = 5.419, *p* = 0.005; *F*_3, 24_ = 5.632, *p* = 0.005 respectively], suggesting within-session habituation in these two groups.

**Table 1:** Raw locomotor activity counts under non-injection control conditions.

**Drug**	**Mean**	**SEM**	**Interval**	**Mean**	**SEM**
**Levorphanol**			**(-)-Pentazocine**		
Interval 1	110.7	7.6	Interval 1	79.2	7.9
Interval 2	100.3	10.7	Interval 2	79.4	6.4
Interval 3	114.6	11.1	Interval 3	74.3	7.2
Interval 4	101.8	9.1	Interval 4	76.0	9.4
**Spiradoline**			**(-)-Metazocine**		
Interval 1	87.0	5.4	Interval 1	79.8	6.2
Interval 2	96.1	7.3	Interval 2	88.0	10.4
Interval 3	92.4	9.2	Interval 3	88.8	8.3
Interval 4	98.5	7.6	Interval 4	86.7	6.6
**Naloxone**			**(-)-Cyclazocine**		
Interval 1	91.5	7.6	Interval 1	64.7	5.1
Interval 2	92.6	5.7	Interval 2	61.6	3.0
Interval 3	95.8	10.2	Interval 3	58.3	3.8
Interval 4	99.4	5.6	Interval 4	60.0	4.1
**Buprenorphine**			**(-)-NANM**		
Interval 1	62.3	4.4	Interval 1	75.9	7.9
Interval 2	58.9	4.9	Interval 2	78.0	7.2
Interval 3	56.3	4.4	Interval 3	69.7	6.7
Interval 4	55.4	2.6	Interval 4	72.8	5.6
**Butorphanol**			**Nalorphine**		
Interval 1	72.8	7.0	Interval 1	86.4	8.8
Interval 2	64.0	4.8	Interval 2	81.3	7.3
Interval 3	57.3	8.1	Interval 3	82.5	10.8
Interval 4	56.6	7.3	Interval 4	76.8	7.6
**Nalbuphine**	**Mean**	**SEM**			
Interval 1	73.4	6.3			
Interval 2	75.8	6.9			
Interval 3	58.2	4.8			
Interval 4	58.1	7.3			

### Levorphanol, Spiradoline, and Naloxone

**Figure 2:**
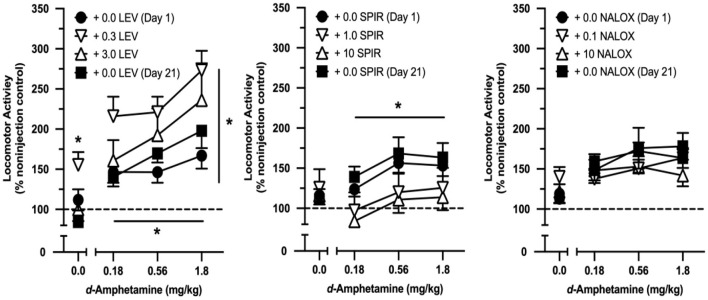
Effects of cumulative doses of *d*-amphetamine when tested alone and when tested in combination with selected doses of levorphanol (LEV; *n* = 10), spiradoline (SPIR; *n* = 10), and naloxone (NALOX; *n* = 10). Vertical axes reflect locomotor activity expressed as a percentage of non-injection control values. Horizontal axes reflect dose of *d*-amphetamine in mg/kg. Points above “0” represent the effects of vehicle (saline) and various doses of opioids tested alone. Vertical lines represent the SEM; where not indicated, the SEM fell within the data point. Single asterisk indicates significant effect of opioid alone. Asterisk with horizontal line indicates significant effect of amphetamine dose. Asterisk with vertical line indicates significant effect of opioid pretreatment.

The selective, high-efficacy mu agonist, levorphanol, increased locomotor activity when administered alone during the first interval of the drug combination tests [*F*_2, 18_ = 4.580, *p* = 0.025]. This effect was biphasic at the two doses tested, with the low (0.3 mg/kg) but not the high (3.0 mg/kg) dose increasing locomotor activity relative to saline (Figure 2). *D-*amphetamine dose-dependently increased locomotor activity [*F*_2, 18_ = 4.307, *p* = 0.030], and this effect was increased by levorphanol [*F*_2, 18_ = 4.215, *p* = 0.032]. The low dose of levorphanol increased the effects of *d*-amphetamine in a greater-than-additive manner [*F*_1, 9_ = 17.124, *p* = 0.003], whereas the effects of a high dose conformed to an effect-additive model. There was no change in the locomotor effects of *d*-amphetamine alone due to repeated testing (planned comparison of Day 1 vs. Day 21: no main effect of day or day x dose of *d*-amphetamine interaction).

The selective, high-efficacy kappa agonist, spiradoline, did not alter locomotor activity when administered alone (Figure 2). *d*-Amphetamine increased locomotor activity in drug combination tests [*F*_2, 18_ = 6.351, *p* = 0.008), but neither dose of spiradoline altered the effects of *d*-amphetamine relative to saline. Similar to that observed in levorphanol-treated rats, there was no change in the locomotor effects of *d*-amphetamine alone due to repeated testing.

The non-selective opioid antagonist, naloxone, did not alter locomotor activity when administered alone and functionally blocked *d*-amphetamine-induced increases in locomotor activity (Figure 2). Moreover, the effects of *d*-amphetamine did not differ from Day 1 to Day 21.

### Intermediate-Efficacy Opioids

**Figure 3:**
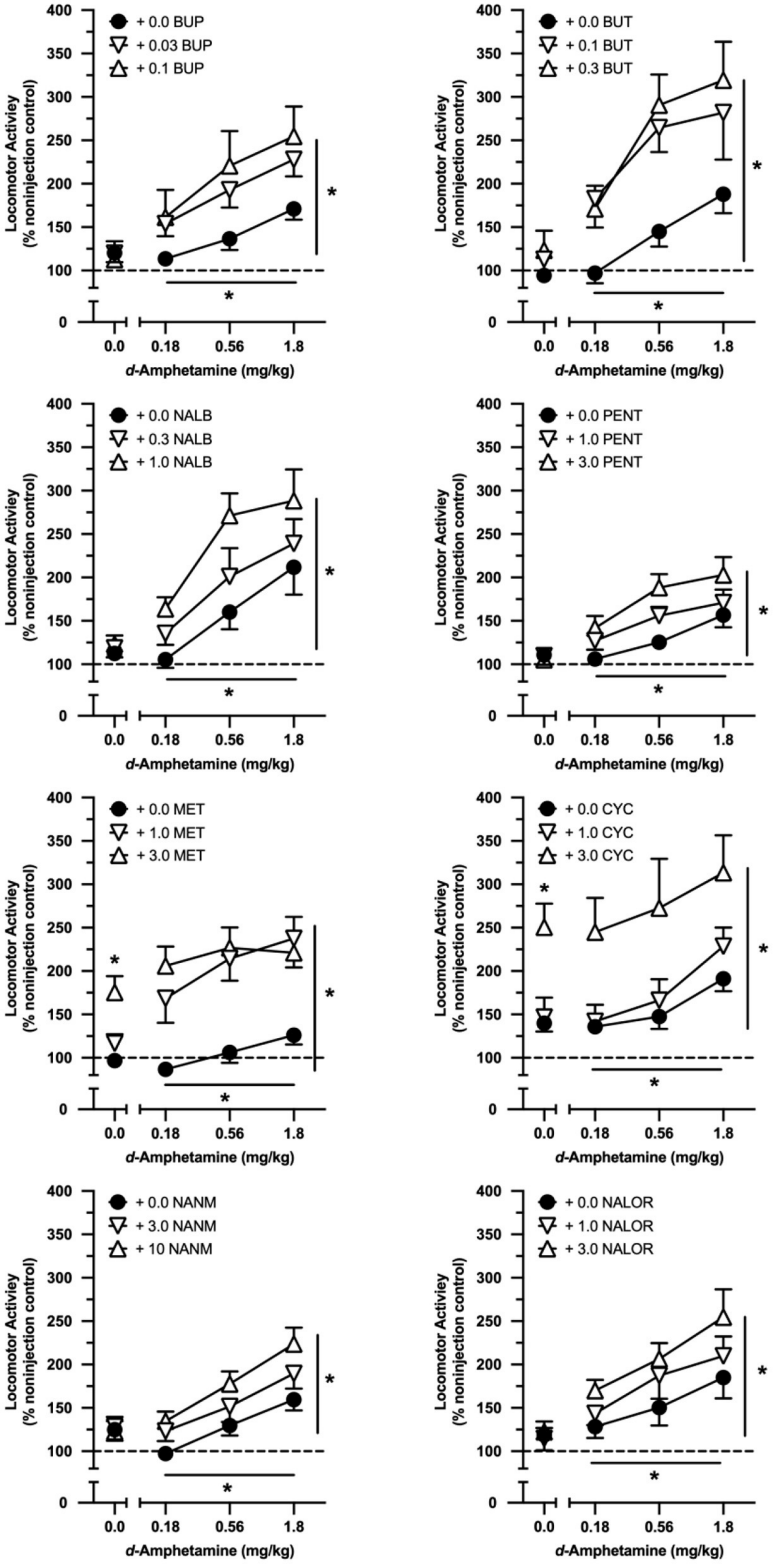
Effects of cumulative doses of *d*-amphetamine when tested alone and when tested in combination with selected doses of buprenorphine (BUP; *n* = 9), butorphanol (BUT; *n* = 10), nalbuphine (NALB; *n* = 9), (-)-pentazocine (PENT; *n* = 10), (-)-metazocine (MET; *n* = 10), (-)-cyclazocine (CYC; *n* = 10), (-)-NANM (NANM; *n* = 10), and nalorphine (NALOR; *n* = 10). Vertical axes reflect locomotor activity expressed as a percentage of non-injection control values. Horizontal axes reflect dose of *d*-amphetamine in mg/kg. Points above “0” represent the effects of vehicle (saline) and various doses of opioids tested alone. Vertical lines represent the SEM; where not indicated, the SEM fell within the data point. Single asterisk indicates significant effect of opioid alone. Asterisk with horizontal line indicates significant effect of amphetamine dose. Asterisk with vertical line indicates significant effect of opioid pretreatment.

In drug combination tests with eight intermediate-efficacy opioids, *d*-amphetamine significantly increased locomotor activity regardless of the opioid administered (see Table 2 for a full ANOVA table listing all significant effects for tests conducted with the intermediate-efficacy opioids). All intermediate-efficacy opioids significantly increased the locomotor effects of *d*-amphetamine (Table 2, Figure 3). The doses of opioids tested varied in their locomotor effects when administered alone, and whether they increased the effects of *d*-amphetamine in an additive or greater-than-additive manner.

**Table 2:** ANOVA table for intermediate-efficacy opioids.

**Drug**	**Pretreatment (opioid alone)**	**Drug combination**		**Effect-additive model**	
		**Opioid dose**	**d-Amp dose**	**Opioid: low**	**Opioid: high**
**Buprenorphine**					
df_factor_, df_error_		2, 16	2, 16	1, 8	1, 8
*F*		15.889	3.700	12.56	13.38
*P*	*NS*	<0.001	0.048	0.008	0.006
**Butorphanol**					
df_factor_, df_error_		2, 18	2, 18	1, 9	1, 9
*F*		6.943	18.503	13.836	15.36
*P*	*NS*	0.006	<0.001	0.005	0.004
**Nalbuphine**					
df_factor_, df_error_		2, 16	2, 16		1, 8
*F*		10.862	31.093		44.533
*P*	*NS*	0.001	<0.001	*NS*	<0.001
**(-)-Pentazocine**					
df_factor_, df_error_		2, 18	2, 18		1, 9
*F*		12.058	7.996		19.223
*P*	*NS*	<0.001	0.003	*NS*	0.002
**(-)-Metazocine**					
df_factor_, df_error_	2, 18	2, 18	2, 18	1, 9	1, 9
*F*	24.378	3.658	62.645	31.903	9.453
*P*	<0.001	0.046	<0.001	<0.001	0.013
**(-)-Cyclazocine**					
df_factor_, df_error_	2, 18	2, 18	2, 18		
*F*	14.115	15.329	7.534		
*P*	<0.001	<0.001	0.004	*NS*	*NS*
**(-)-NANM**					
df_factor_, df_error_		2, 18	2, 18		1, 9
*F*		14.41	11.82		51.517
*P*	*NS*	<0.001	0.001	*NS*	<0.001
**Nalorphine**					
df_factor_, df_error_		2, 18	2, 18	1, 9	1, 9
*F*		18.972	7.257	13.22	8.134
*P*	*NS*	<0.001	0.005	0.005	0.019

*NS indicates non-significant main effect. No significant interactions for the drug combination data (opioid dose × d-amphetamine dose) or the model data (model × d-amphetamine dose) were obtained. In the 2 × 2 ANOVA for model, a main effect for dose of d-amphetamine was observed under all conditions but are not shown in the table*.

Neither dose of buprenorphine, butorphanol, nalbuphine, (-)-pentazocine, (-)-NANM, or nalorphine increased locomotor activity when administered alone; however, all six intermediate-efficacy opioids increased the effects of *d*-amphetamine (Table 2, Figure 3). All six opioids increased the effects of *d*-amphetamine in a greater-than-additive manner at the higher test dose, whereas only buprenorphine, butorphanol, and nalorphine increased the effects of *d*-amphetamine in a greater-than-additive manner at the lower test dose. In all cases, opioid-induced increases in *d*-amphetamine's locomotor effects were dose-dependent and quantitatively greater at the higher than lower test dose of the opioid.

(-)-Cyclazocine and (-)-metazocine dose-dependently increased locomotor activity when tested alone, and both drugs significantly increased the effects of *d*-amphetamine in a dose-dependent manner (Table 2, Figure 3). Both doses of (-)-metazocine increased the effects of *d*-amphetamine in a greater-than-additive manner, whereas both doses of (-)-cyclazocine conformed to an effect-additive model.

## Discussion

The principal finding of this study is that eight structurally and pharmacologically diverse intermediate-efficacy opioids increased the effects of *d*-amphetamine in a manner that was generally similar to the selective, high-efficacy mu agonist, levorphanol. The only opioids that failed to increase the effects of *d*-amphetamine were the selective, high-efficacy kappa agonist, spiradoline, and the non-selective opioid receptor antagonist, naloxone. The failure of spiradoline to enhance *d*-amphetamine's locomotor effects suggests that the effects of the intermediate-efficacy opioids were not mediated by the kappa receptor. Moreover, the finding that naloxone prevented *d*-amphetamine-induced locomotor activity suggests that mere occupation of opioid receptors is not sufficient to enhance *d*-amphetamine-induced locomotion. Together, these data suggest that agonist activity at the mu receptor is likely responsible for the ability of intermediate-efficacy opioids to increase the locomotor effects of d-amphetamine.

The intermediate-efficacy opioids tested vary in structure, with multiple morphinans (e.g., levorphanol, butorphanol, nalorphine) and benzomorphans [e.g., (-)-pentazocine, (-)-metazocine, (-) cyclazocine] represented. Moreover, these opioids differ in their relative selectivity for mu vs. kappa receptors, and included both mu-preferring (e.g., buprenorphine) and kappa-preferring [e.g., (-)-pentazocine] opioids (33, 34). Most importantly, the opioids differ in their relative efficacy at the mu receptor, with an estimated rank order of levorphanol > buprenorphine > butorphanol ≥ nalbuphine > (-)-metazocine ≥ (-)-pentazocine ≥ (-)-cyclazocine ≥ nalorphine > naloxone (29–31).

These findings are consistent with a previous study demonstrating that many of these same opioids increase the effects of cocaine under similar conditions (28). In that study, all intermediate-efficacy opioids except nalorphine (i.e., the opioid with the lowest estimated relative efficacy at the mu receptor of those tested) increased the effects of cocaine. Similar to the present study, the ability of an intermediate-efficacy opioid to increase the effects of cocaine was shared by levorphanol, but not by spiradoline or naloxone. The concordance between these studies demonstrates that the effect of opioids on stimulant-induced locomotion are consistent across stimulants with different mechanisms of actions (i.e., dopamine releasing agent vs. dopamine reuptake inhibitior).

*d-*Amphetamine-induced locomotor activity is mediated by the release of striatal dopamine, primarily in the nucleus accumbens. The cell bodies of dopamine-releasing nerve terminals in the nucleus accumbens are located in the ventral tegmental area (VTA). These dopamine-releasing neurons are under tonic inhibitory control by GABAergic neurons also located in the VTA. These GABAergic neurons, in turn, are under tonic inhibitory control by endogenous opioid peptides that bind to mu receptors on the cell surface. Activation of these mu opioid receptors by mu receptor agonists represents one mechanism by which high-efficacy mu agonists increase the locomotor effects of psychomotor stimulants (35). In general, opioid antagonists are more effective in blocking the effects of dopamine releasers like amphetamine than reuptake inhibitors like cocaine [e.g., (36)]. These findings have been interpreted to suggest that endogenous opioid release may contribute to some effects of *d-*amphetamine, which has several implications for the present study.

One implication of the present findings is that the endogenous tone of these mu receptors is low, given that opioids possessing very low efficacy at the mu receptor were able to increase the effects of *d*-amphetamine in either an additive or greater-than-additive manner. A second and similar implication is that the enhancement of *d-*amphetamine-induced locomotion by opioids has a very low efficacy requirement, and this assay provides a very sensitive endpoint of mu-opioid activation. Additional studies showing the effects of these intermediate-efficacy opioids are reversible with mu-selective neutral antagonists would offer additional support for this possibility.

We have presented evidence that intermediate-efficacy mu opioids increase the locomotor effects of both a dopamine releaser (i.e., *d*-amphetamine; present study) and a dopamine reuptake inhibitor [i.e., cocaine (28)]. The only relevant difference between these studies is that the very low efficacy mu agonist nalorphine increased the locomotor effects of *d-*amphetamine at doses that did not alter the locomotor activity of cocaine. We are hesitant to make cross-study comparisons across studies conducted years apart, but it is notable that the locomotor effects of both drugs were very sensitive to opioid administration. Consequently, one final implication of these data is that intermediate-efficacy mu opioids can increase stimulant-induced locomotor activity under conditions that are dependent on neuronal activity and cell firing (in the case of the reuptake inhibitor, cocaine) and under conditions that are independent of neuronal activity and cell firing (in the case of the dopamine releaser, *d-*amphetamine).

Several limitations of the present study should be acknowledged. First, the study only used male rats, and we emphasize that future studies must be conducted in females to test the hypothesis that these findings can be generalized across biological sex. Second, the study only measured locomotor activity for 120 s, which is much shorter than most studies examining locomotor activity that measure behavior for 60 min or longer. Our time-course data mitigates this concern to some extent, showing that the effects observed during 2-min “snapshots” are similar to those obtained over extended and continuous testing periods [e.g., (37, 38)]. Third, only two doses of each opioid were tested. Although at least one dose of each opioid increased the effects of *d*-amphetamine, some opioids did not alter locomotor when administered alone at the doses tested. Testing a wider dose range would reveal whether higher (or lower) doses would increase locomotor activity in the absence of *d*-amphetamine. Finally, drug interactions were quantified using an effect-additive approach. This approach has several limitations relative to a dose-additive approach (39), and any conclusions regarding “synergistic” interactions between opioids and d-amphetamine should be made with an abundance of caution.

The translational relevance of this study is that intermediate-efficacy opioids with diverse chemical and pharmacological properties all increase the effects of *d*-amphetamine, including those opioids with very low efficacy at the mu receptor. These findings imply that potentially problematic dopamine-mediated effects may be observed in recreational and clinical settings when these drugs are combined. Similar to locomotor activity, the abuse-related effects of mu opioids and *d-*amphetamine are mediated by dopaminergic activity in the nucleus accumbens. Consequently, substitution of high-efficacy mu agonists for lower-efficacy agonists may not mitigate the abuse liability of these opioid-stimulant drug combinations.

## Data Availability Statement

The raw data supporting the conclusions of this article will be made available by the authors, without undue reservation.

## Ethics Statement

The animal study was reviewed and approved by Davidson College Animal Care and Use Committee.

## Author Contributions

MS conceived of the study, analyzed the data, and wrote the manuscript. SB, CB, SB, AC, LC, MF, AG, AJ, DL, AM, CM, IR, JR, JS, MW, and SY collected the data. All living authors approved the final draft and are accountable for the work.

## Funding

This work was supported by the National Institutes of Health (Grant Numbers: DA045364 and DA0317254).

## Conflict of Interest

The authors declare that the research was conducted in the absence of any commercial or financial relationships that could be construed as a potential conflict of interest.

## Publisher's Note

All claims expressed in this article are solely those of the authors and do not necessarily represent those of their affiliated organizations, or those of the publisher, the editors and the reviewers. Any product that may be evaluated in this article, or claim that may be made by its manufacturer, is not guaranteed or endorsed by the publisher.
